# Evaluating mob stocking for beef cattle in a temperate grassland

**DOI:** 10.1371/journal.pone.0226360

**Published:** 2019-12-12

**Authors:** Benjamin F. Tracy, Robert B. Bauer

**Affiliations:** School of Plant and Environmental Sciences, Virginia Tech, Blacksburg, Virginia, United States of America; Universidade Federal de Mato Grosso do Sul, BRAZIL

## Abstract

Mob stocking is a type of livestock management method where high densities of animals are restricted to a small area of grassland for short periods of time (e.g., 12–24 hr.) before being moved to new forage. Use of mob stocking has generated considerable interest among forage-livestock professionals in recent years, but questions remain about its purported benefits to cattle and forage plants. To address questions about the possible benefits of mob stocking, a 3-yr study (2014–2016) was conducted in Virginia, USA comparing mob, rotational, and continuous stocking methods in a temperate grassland common to that region. The main objective of this study was to evaluate how mob-stocking management affected selected forage variables, cattle performance, and legume/weed abundance. Herbage mass and nutritive value were measured monthly. Cow and calf weights and body condition score (BCS) were used as indicators of animal performance. Legumes (red and white clover) were over-seeded prior to the study, and their abundance along with weeds were evaluated annually thereafter. Mean herbage mass and forage nutritive values were similar across mob, rotational and continuously stocked systems despite extra-long rest periods that allowed grasses to grow tall and over-mature under mob stocking. Cow weights going into winter were lower (*P* < 0.05) under mob stocking (619 kg) compared with continuous stocking (688 kg) possibly because many tall grasses were trampled and not grazed. Lastly, we found mob stocking can favor establishment of erect-growing red clover (*Trifolium pretense* L.), but it had no effect on weed abundance. Overall, we found few compelling reasons why mob stocking should be adopted for season-long forage and livestock production over other stocking methods in this environment.

## Introduction

Mob stocking is a type of rotational or managed grazing that involves intensive decision-making to control livestock stocking rates and forage removal to produce desired outcomes [1]. Mob stocking can be difficult to define, but it usually involves restricting a high density of animals (e.g., 50,000 to over 200,000 kg BW ha^-1^) to a paddock before being moved to new forage usually within 24 hours ([2–4]). Mob stocking tends be initiated on tall, mature forage followed by long recovery periods–usually 90 d or longer. Under such conditions, paddocks may be grazed just once or twice per season and many paddocks are required for the rotation sequence. During the long rest periods from grazing, forage accumulates and typically becomes over-mature. More moderate rotational stocking also uses recurring periods of grazing to match forage growth but employs fewer paddocks. Rotational stocking is similar in principle to mob stocking except stocking density is lower and pasture recovery periods are shorter (e.g., 15–30 d). Continuous stocking is a more most common management method and involves no or minimal movement of livestock among different paddocks [1].

Mob stocking was promoted by Allan Savory in the 1980s as part of a more holistic approach to rangeland management [5] and has generated considerable interest in the forage-livestock community [6, 7, 8]. Anecdotal observations have described the benefits of mob stocking methods. Practitioners who adopt mob stocking have reported increased forage production, higher plant diversity, fewer weeds, improved distribution of livestock grazing and nutrient distribution from waste, and better soil health [7]. Although many studies have compared rotational with continuous stocking methods [9, 10, 11, 12], less comparative research has been done on mob stocking approaches. In this study, our main goal was to collect replicated field data to evaluate the potential benefits of mob stocking in a temperate grassland. Results reported here were from a 3-yr. study conducted from 2014 to 2016. The main objective of the study was to compare herbage mass, herbage nutritional value, and animal performance across three stocking methods classified as mob, rotational and continuous. A second objective sought to evaluate how mob stocking would affect plant species composition–specifically legume and weed abundances.

## Materials and methods

### Study site

A replicated grazing experiment was conducted at the Virginia Tech Shenandoah Valley Agriculture Research and Extension Center (SVAREC) from 2014–2016. The study site was located in central Virginia (37°55’45” N latitude and 79°13’11” W longitude, elevation: 582 m) and consisted of 90 ha of pastureland. The soils at the site were classified as well-drained Frederick and Christian gravelly silt loams, with slopes ranging from 2% to 20%. Soils across the site were tested for pH, P and K prior to the start of the trial and fertility was adjusted on experimental units based on recommendations by the Virginia Tech Soil Testing Lab. Vegetation was dominated by endophyte-infected tall fescue [*Schedonorus arundinaceus* (Schreb.) Dumort.], orchardgrass (*Dactylis glomerata* L., Kentucky bluegrass (*Poa pratensis* L.). In February 2007 and 2009, 4.5 kg ha^-1^ of white clover (*Trifolium repens* L.) and red clover seed, equally proportioned, were broadcast over the experimental area. The study site has a humid continental climate with 993 mm average annual precipitation, and average monthly temperatures range from 0.3°C in January to 23.1°C in July. Temperature and precipitation data reported in this paper were taken at a weather station located on the site.

### Stocking methods and experimental design

The experiment consisted of a randomized arrangement of mob, rotational, and continuous stocking treatments. From May 2014 to November 2016, stocking treatments were applied to nine, 6.4-ha pastures across the site to achieve three replications of each. Within treatment areas, pastures were subdivided into paddocks of equal area with high-tensile wire and polywire to implement the stocking treatments. At the beginning of the study, beef cows from the SVAREC herd were assigned to treatments to ensure stocking rate was similar across stocking treatments. Mature beef cows (623 ± 7 kg) were stocked in all treatments at 11.5 AUM ha^-1^ (Table 1), and calves (35 ± 2 kg) born in fall were retained with their dams post-partum. Mob stocking consisted of three stocking periods each year on 64, 0.1-ha paddocks that were allocated to cattle every 24 h. Mob-stocked paddocks were created using single-strand electric fence to provide 0.1- ha strips for grazing as cattle were moved through a series of nine pre-existing 0.7 ha pastures within each experimental unit. Mob-stocked paddocks were not back-fenced to allow cattle access to water at a fixed location on one end of the pasture. A stocking density of approximately 50,000 kg live BW ha^-1^ was maintained on the paddocks, which would be considered a lower density for most mob stocking situations. Rotational stocking was applied across eight, 0.8-ha paddocks using stocking periods of 4 d with a fixed 28 d rest period for each paddock. Continuous stocking consisted of one uninterrupted stocking period that spanned ~200 d beginning in early May. The seasonal grazing duration for mob and rotational treatments was the same as the continuous treatment. Cows were bred to give birth in fall. Cows and calves remained in the experimental units over the winter and were fed hay until May. Cows and calves in rotational and mob systems were confined to a 0.7 or 0.8-ha hay-feeding area, during winter. In the continuous system, hay was fed within a single confined area but cows and calves had access to the entire 6.5-ha pasture. Grazing, with cows only, began again in early May.

**Table 1:** Herd characteristics and stocking management during the course of the study. Values are averages from 2014–2016.

Stocking Method	Grazing days (d)	Cattle paddock^-1^	Body weight (kg)	Paddocks system^-1^	Paddock area (ha)	Stocking density *AU ha^-1^	Stocking rate ^#^AUM ha^-1^
**Mob**	200	8	621	64	0.1	109	11.5
**Rotational**	200	8	625	8	0.8	14	11.5
**Continuous**	200	8	625	1	6.4	1.7	11.5

*AU: animal unit (454 kg live BW),

^**#**^AUM: animal unit month

### Herbage mass

Standing herbage biomass was harvested in 2014 and 2015, but not 2016 due to limited resources. Herbage was harvested at month intervals by cutting all vegetation within 9–10 randomly located areas within each experimental unit (6.4 ha). In each sampling area, a 0.7 x ~4 m swath was cut 2 cm above the soil surface with a mechanical harvester. The fresh herbage from each swath was weighed on scale built into the harvester and sub samples (~200 g) were dried in a force-draft oven at 55°C for 48 h. The sub samples were used to calculate the dry weight of herbage mass and estimate forage nutritive value described below. For rotational and mob treatments, herbage mass samples were collected across experimental units without regard to location of cattle in the rotation sequence. Sampling in this manner included grassland areas at various stages of regrowth from recently grazed to ungrazed areas. This method was used to provide a herbage mass ‘inventory’ each month for the respective stocking treatments. It was considered the fairest way to compare herbage mass dynamics among these very different stocking treatments. In 2015, a scale malfunction caused some irregularities in the herbage mass data collected in the late season so we elected to exclude that year from the statistical analysis.

### Nutritive value

Oven-dried samples of standing herbage biomass were milled with Thomas-Wiley mills (2-mm screen; Philadelphia, PA) and cyclone mills (1 mm screen; Cyclotec, Hilleroed, Denmark) and then scanned with near infrared reflectance spectroscopy (NIRS; FOSS 6500, Hilleroed, Denmark). Crude protein (CP), acid detergent fiber (ADF), and neutral detergent fiber (NDF) concentrations in herbage samples were predicted from calibration equations developed previously at Virginia Tech from tall fescue-based pasture samples (n = 136). The standard error of calibration (SEC), the coefficient of determination (R^2^) for the respective NIRS equations were as follows: CP: 0.93 and 0.96, ADF: 1.65 and 0.93, NDF: 2.62 and 0.94, respectively. Cross-validation statistics did not differ appreciably from calibration statistics. Accuracy of the equations also was assessed with the Global H (Mahalanobis distance) and Neighborhood H statistics [13]. Nutritive value analysis was not completed for samples collected in September 2015.

### Cattle weights

Cow weights were taken at the end of the grazing season when cows were bred in late November-early December (2014–2016). Body condition score (BCS) of each cow was taken at this time using a 1–9 scale where 1 is extremely thin and 9 is obese [14]. A BCS between 5–7 is considered ideal for beef cows. In addition to cow weights, calves were weighed at birth in the fall, and at weaning in May. An adjusted 205-d weaning weight was used to compare stocking treatments.

### Plant species composition: Clover and weed cover

Percent ground cover of herbage, dead material, and bare soil was visually estimated in 0.5-m^2^ rectangular quadrats each May before grazing began. A modified Daubenmire method was used to estimate visually the percent ground cover of plant species [15, 16]. Herbage cover measurements were made at 20–25 sampling points per experimental unit. Weed species cover was calculated from sum of ‘non-forage’ plants that excluded common forage species like bluegrass, orchardgrass, and tall fescue.

### Statistical analysis

Statistical analysis was performed in *R* Version 3.2.1 [17]. Herbage mass and nutritive value data were analyzed using ANOVA including treatment, month, and year (for nutritive value only) as main effects and their respective interactions. Subsamples within each experimental unit were averaged prior to analysis to generate a single value for each of the respective variables. Treatments were assessed for homogeneity of variance and normality. Treatment differences within years or months were evaluated separately if interactions were statistically significant (*P* < 0.05). Tukey’s Honestly Significant Difference (HSD) was used to determine mean separation for significant main effects (α = 0.05). Cattle variables and plant species composition also were analyzed using ANOVA with year, treatment and their interaction as main effects. Subsamples were averaged within each replicate before subjecting data to ANOVA Additionally, for data collected in 2014, we used regression to evaluate whether the date of grazing initiation on mob and rotationally stocked paddocks predicted herbage mass later in the grazing season.

## Results

### Weather variables

Growing season precipitation (April-October) in 2015 exceeded the 30-yr. average while precipitation in 2014 and 2016 was below average (Table 2). Excluding October 2016, we observed no sustained nor exceptionally dry periods lasting more than 30 d during the respective growing seasons. Growing season air temperatures were at or above long-term means.

**Table 2:** Growing season precipitation at the study site from 2014 to 2016 and the 30-yr. mean for each respective month.

	Precipitation (mm)			
Month	2014	2015	2016	30-yr. average
**April**	85	112	34	80
**May**	92	66	113	95
**June**	114	93	117	92
**July**	55	135	37	99
**August**	77	26	56	92
**September**	13	133	164	96
**October**	118	77	0.3	76
**Average**	79	92	74	90

### Herbage mass

**Fig 1:**
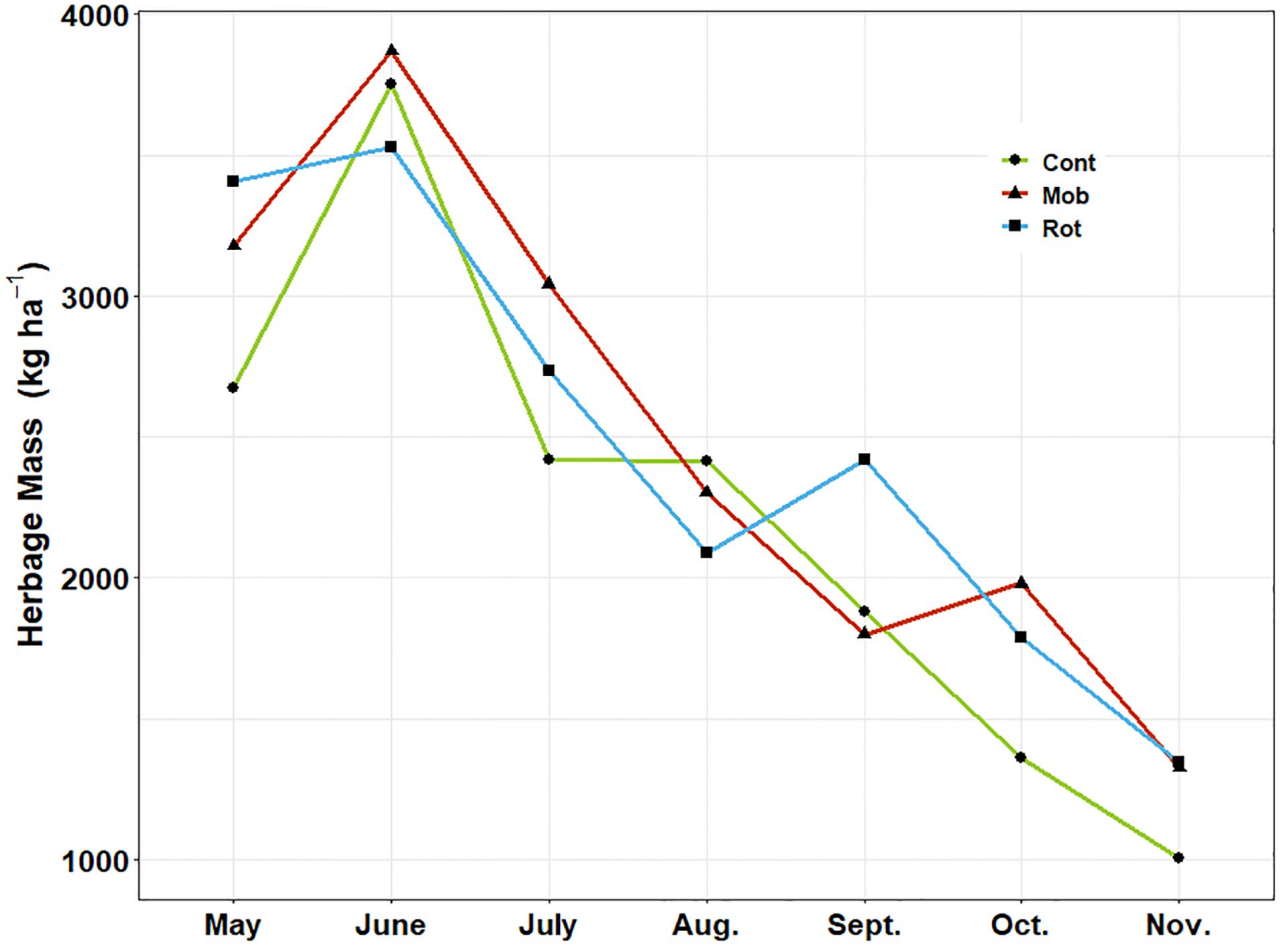
Mean herbage mass averaged over the 2014 growing season. CONT, MOB and ROT labels refer to continuous, mob and rotational stocking treatments, respectively.

Herbage mass data was analyzed only for the 2014 growing season. Temporally, herbage mass in all paddocks increased from 3,363 kg ha^-1^ in May to 4,145 kg ha^-1^ in June but subsequently decreased during the June to November period (Fig 1). Herbage mass did not differ among stocking treatments (*P* < 0.05). In 2014, the date of grazing initiation in mob grazed paddocks was significant as a predictor of herbage mass later in the growing season (R^2^ = 0.35, P = 0.001). Mob grazed paddocks averaged 28 kg ha^-1^ more standing herbage each day after mob stocking was initiated in May. Herbage mass at subsequent dates in rotationally grazed paddocks was not significantly predicted by date of grazing initiation (*P* = 0.36).

### Nutritive value

**Fig 2:**
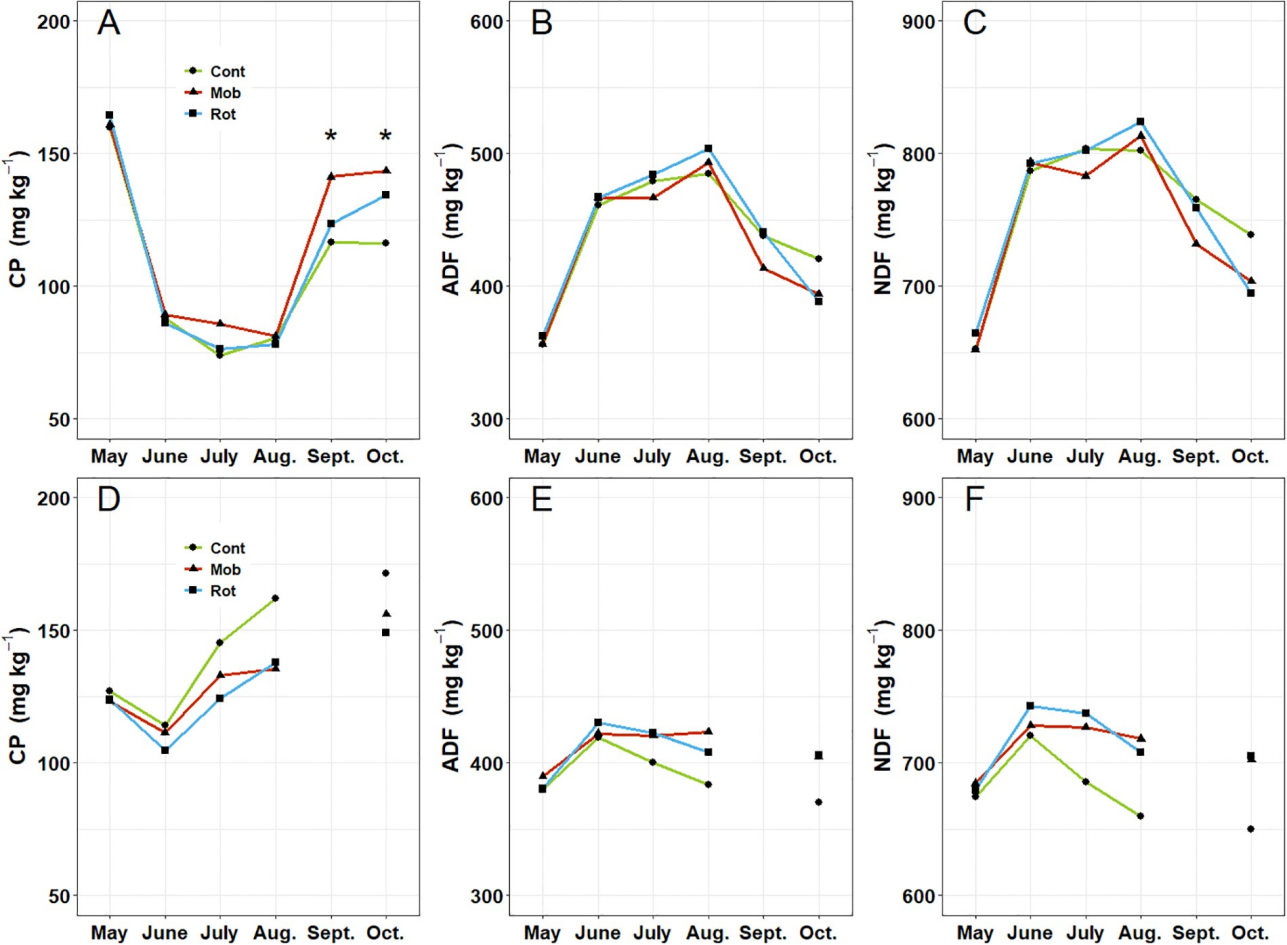
Mean crude protein (CP), acid detergent fiber (ADF) and neutral detergent fiber (NDF) concentrations in herbage from 2014 (A, B, C) and 2015 (D, E, F) growing seasons. CONT, MOB and ROT labels refer to continuous, mob and rotational stocking treatments, respectively. * indicates statistical difference among treatments (P < 0.05) for the respective month.

A significant year x month interaction was noted for all nutritive value indices so years (2014 and 2015) were analyzed separately. In 2014, nutritive value indices (CP, ADF and NDF) of herbage varied predictably with the growth of cool-season plants. For example, crude protein (CP) was greatest in spring when plant growth was most vigorous, least in hot mid-summer months, and intermediate in fall (Fig 2A). Both fiber components (ADF and NDF) showed significant month effects (P < 0.01) but did not differ among treatments. Fiber concentrations were lowest in spring, highest in summer and intermediate in fall (Fig 2B and 2C). The interaction of stocking method x month was marginally significant (P = 0.065) for CP in 2014. Crude protein content of herbage was similar among treatments until fall when the mob-stocked treatments exceeded the continuously grazed systems (Fig 2A, Tukeys HSD test, α = 0.05, df = 12).

Different seasonal patterns were noted for nutritive value indices in 2015. Crude protein increased more in the continuous stocking treatments as the growing season progressed compared with other treatments (Fig 2D, month x treatment interaction, *P* = 0.07). Fiber concentrations also showed significant month by treatment interactions (*P* < 0.01). Concentrations were similar in spring among the treatments but ADF and NDF in continuously- stocked treatments declined more as the season progressed compared with mob and rotational treatments (Fig 2E and 2F).

### Cattle

From 2014 to 2016, cow weights and BCS at breeding were significantly lower in the mob-stocking treatment compared with the continuous-stocking treatment (Table 3). Calf birth weights were lowest in the rotational treatment and similar in mob and continuous treatments. Calf weaning weights also differed among systems and were highest in the continuous-stocking treatment (Table 3).

**Table 3:** Cattle measurements across years (2014 to 2016) and stocking treatments. CONT, MOB and ROT labels refer to continuous, mob and rotational stocking treatments, respectively. SE is 1 standard error.

Year	Treatment	Cow wt. (kg)	Cow Body Condition Score (1–9)	Calf Birth Wt. (kg)	Calf Wean Wt. (kg)
**2014**	**CONT**	**643**	**6.5**	**37**	**195**
**2015**	**CONT**	**712**	**7.6**	**39**	**205**
**2016**	**CONT**	**710**	**7.8**	**36**	**230**
**2014**	**MOB**	**626**	**5.8**	**36**	**187**
**2015**	**MOB**	**618**	**6.0**	**38**	**182**
**2016**	**MOB**	**614**	**6.4**	**38**	**203**
**2014**	**ROT**	**619**	**6.1**	**33**	**194**
**2015**	**ROT**	**657**	**6.7**	**35**	**193**
**2016**	**ROT**	**637**	**7.0**	**33**	**196**
***Means***†	***CONT***	***688 a***	***7*.*3 a***	***38 a***	***210 a***
	***MOB***	***619 b***	***6*.*1 b***	***37 a***	***191 b***
	***ROT***	***638 b***	***6*.*6 b***	***34 b***	***195 b***
	***SE***	***7*.*8***	***0*.*14***	***0*.*50***	***3***
	***P-value***	***<0*.*001***	***<0*.*001***	***<0*.*001***	***0*.*003***

^†^ Treatment means (italics) within each column followed by the same letter do not differ (Tukey’s Honestly Significant Difference Test, α = 0.05, n = 27).

### Clover and weed cover

**Fig 3:**
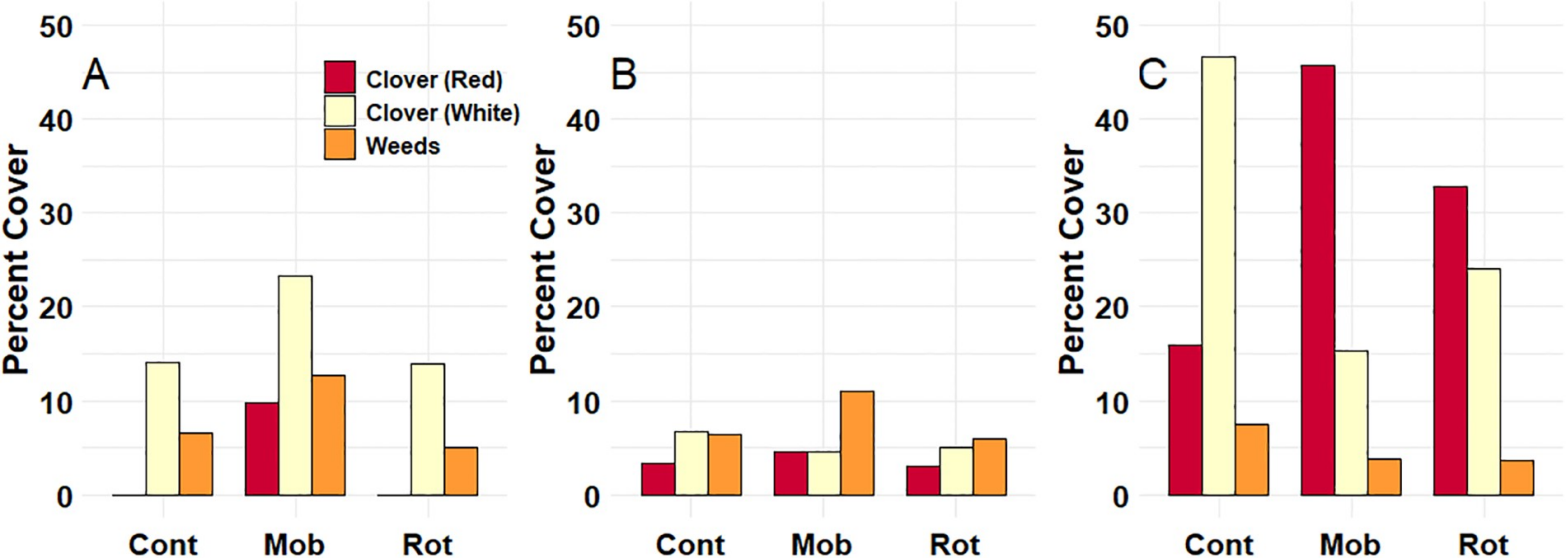
Percent ground cover (%) of clovers (red and white) and weeds in May 2014 (A), 2015 (B), and 2016 (C) before grazing was initiated each season.

Clover seed was overseeded across all experimental units prior to start of this study. White clover, red clover, and weed cover all showed significant year x treatment interactions (*P* < 0.01). The interaction was largely a function of the 2016 growing season, when red and white clover cover was much greater compared with the first two years (Fig 3A, 3B and 3C). By the start of 2016 season, continuous stocking had clearly favored white clover, while mob and rotational stocking promoted more red clover (Fig 3C). In 2014, red clover was only present in mob-stocked treatments. Weed cover showed no consistent trend with stocking treatment (Fig 3A, 3B and 3C).

## Discussion

### Herbage mass

The amount of herbage mass measured in 2014 did not differ among the stocking treatments. Given that stocking rate was the same across treatments, this result suggests overall forage growth or net primary production probably was not affected by the different stocking methods that year. Overall trends showed a general decline in herbage mass over the growing season (Fig 1), which should be expected. Although herbage mass could not be reported from the 2015 and 2016 growing seasons, our observations suggested that the seasonal trends would have been similar to 2014. Interestingly, we found that early mob stocking tended to reduce subsequent herbage production later in the season. Rotationally grazed systems, however, did not show the same pattern. Several interrelated factors associated with cattle stocking density may explain the differing responses. For example, grazing intensity early in the season was greater under mob stocking compared with rotational stocking, and this may have stressed grasses more [18]. Defoliation stress may have reduced root biomass and extra carbohydrates mobilized for regrowth may have combined to reduce regrowth potential [19]. Later initiation of grazing has been shown to benefit sward productivity in previous studies of cool-season pastures. Other studies from a similar region found that herbage mass was 33% greater when grazing was initiated in mid-May compared with mid-April on set-stocked pastures of bluegrass and white clover [20]. We suggest that if season-long mob stocking is adopted in a temperate environment like our study site, it may make sense to defer grazing in early spring an extra 10–14 d to allow for more herbage accumulation.

### Nutritive value

In 2014, the nutritive value of herbage biomass varied during the growing season as would be expected based on seasonal changes in plant maturity, soil organic matter mineralization, and cover of grass and dead material in the sward [21]. Interestingly, during the first season, we found higher CP concentrations in fall under mob stocking (Fig 2A). Possibly, greater resources for regrowth with extended pasture rest may have improved the growth status of mob-grazed pastures and allowed them to resume growth more quickly during the cooler fall conditions relative to continuously stocked paddocks that were not rested. Although greater legume abundance might also explain the differences in CP, clover cover was less than 1% in both mob and continuous systems that October [22]. Bauer [22] also evaluated mob stocking at a nearby site and did not find a similar response in nutritive value during fall suggesting our results may have been unique to the site.

Nutritive value indices did not vary as much over the 2015 growing season and tended to improve slightly under continuous stocking as the season went on (Fig 2D–2F). We can only speculate on why this occurred, but it may have been related to vigorous white clover growth that was promoted by a combination of close continuous stocking and high summer rainfall in 2015 (Table 1). A high proportion of white clover in the continuous treatment forage samples could have contributed to the results observed, and this was supported by data collected by Bauer [22]. In July and September 2015, white clover averaged 22% cover in the continuously stocked treatment while mob and rotational treatments averaged 8 and 11%, respectively.

We found few instances where nutritive value indices dropped below acceptable nutritional thresholds for low-maintenance animals like dry cows. For example, herbage intake generally is limited when CP concentrations are less than 60–80 g kg^-1^ DM [23, 24], and mean CP concentrations did not reach these levels. Although many studies have examined the relationship between stocking methods and forage nutritive value, few have concluded that manipulation of rest period or grazing intensity alone can explain variation in the nutritive value of forage across time [25]. More often, observed differences in nutritive value between grazing methods are attributed to differences in stocking rate [25]. Other studies have found no difference in herbage nutritive value between rotational and continuous stocking systems [9, 26, 27].

### Cattle

We found that cows grazing in the mob-stocking treatments were consistently lighter at the end of each grazing season compared with cows under continuous stocking. Lower cattle weights under mob stocking have been shown in some studies [4, 28], while others have found no difference compared with moderate rotational stocking [29]. The weight differences in our study were relative, and BCS scores in mob stocking treatments were still within the ideal range for beef cattle (e.g., 5–7 BCS) [14]. In fact, calf birth weights were similar in mob and continuous treatments and actually lowest under rotational stocking (Table 3). The forage variables we measured and observed could not explain the lower cow weights as forage mass and nutritive value were not consistently lower under mob stocking. Although we did not measure forage mass or nutritive values in 2016, we suspect the mostly favorable growing season weather during that year (Table 1) likely would have produced similar results in forage measurements. Indeed, forage mass and quality probably would have been greater in mob systems, especially considering the ample red clover cover recorded in 2016 (Fig 3). The moderate stocking rates used in this study combined with favorable growing season rainfall and abundant white clover likely benefitted cow performance on continuously stocked treatments.

We hypothesize the lower cow weights in mob relative to continuous-stocking systems could have been a function of several factors. Under mob stocking, cows had access to mostly tall, overly mature grass, of which at least 40% was trampled and likely not available for grazing [22]. Volesky et al. [4)] suggested lower steer weight gain on mob-stocked pastures was due to trampling and resultant lower forage utilization. Since our herbage harvesting methods did not distinguish trampled and upright vegetation, the herbage mass measurements may have overestimated forage that was easily available for cows to graze. In that case, it was possible that the limited available forage could have caused the cows to lose weight in fall. Secondly, the same cow groups were retained within pasture treatments year-round over the entire study. Although cows were initially assigned so that herd weights would be similar across stocking method treatments, cows in the mob-stocking treatments could have differed in some unknown way. The lower birth weights in the rotational system might have been an artifact of this management as well.

The lower calf weaning weights under mob and rotational stocking were probably related to differences in winter hay feeding management. Cows and calves in mob and rotational systems were confined to 0.8 ha hay feeding paddocks in winter while animals in the continuous systems had access to ~ 6 ha of pasture while being fed hay. Although ample hay was available at all times, cows and calves in continuous systems had access to more young, nutritious grass in early spring, and this may have benefitted weaning weight [20].

### Clover and weed abundance

A second objective of this study was to evaluate how mob stocking might affect clover establishment and weed pressure. We predicted that long rest periods between mob stocking events would suppress clover establishment and possibly reduce weed pressure, mostly owing to increased light competition from tall grasses. Overall, weed pressure was not affected by stocking method, but this was not the case for clover establishment. White clover was more favored by continuous stocking in part because the prostrate growth habit of this species makes it tolerant to close grazing [30, 31], which is common under continuous stocking. We also found reasonable white clover abundance in mob-stocking treatments, which was surprising as it should be more intolerant of shading by grasses. A recent study that evaluated the effect of high-density grazing in grass-legume mixtures found that intense grazing reduced persistence of several planted legumes, but that white clover was negatively impacted the least [32]. Erect-growing legumes such as red clover should be able to compete well with grasses for light [33, 34, 35], and we found that red clover did respond positively to mob stocking (Fig 3). Grazing in late fall, and favorable weather conditions also likely helped to promote clover abundance. Schlueter and Tracy [36] found that clover establishment was favored by close grazing in late fall, which removed much of the herbage mass, followed by abundant spring rainfall. In our study, late fall grazing effectively reduced herbage mass to approximately 1200 kg ha^-1^ most years. This level of forage biomass was almost low enough to limit intake by cattle [23] and should have provided ample space for clover species to emerge and grow in early spring. Coincidentally, the highest clover abundance we recorded occurred in May 2016 when precipitation greatly exceeded the long-term mean. We propose that interactions among plant, soil, and weather variables probably had a greater influence on clover or weed abundance than grazing method.

## Conclusions

We evaluated mob-stocking methods for beef cattle in a temperate grassland (Virginia, USA) over three growing seasons. Few advantages to mob stocking were found compared with more typical rotational or continuous stocking management in this environment. The cattle variables were the best indicators to evaluate performance of the grazing treatments, and we found that cows under mob-stocking were consistently lighter than those in continuous-stocking treatments. We hypothesize that cows in the mob-stocking treatments could not obtain sufficient forage late in the season due to trampling of tall grasses and may have lost weight as a consequence. Despite lower weights, cows under mob stocking were still in good body condition for breeding and calf birth weights were not negatively affected. With respect to clover establishment, we found that mob stocking favored erect-statured legumes like red clover especially if spring rainfall was above average. Aside from mob stocking, our results also suggested that low-density rotational stocking systems were not always superior to continuous stocking. Moderate stocking rates used in this study combined with a favorable distribution of growing-season rainfall probably contributed to good performance of the continuous stocking compared with mob and rotational stocking. We conclude that mob stocking probably would be more useful for short-term vegetation management objectives rather than employed in a season-long stocking method. The extra management, infrastructure, and potential land resources needed to carry out long-term mob stocking seems an unwise investment for limited benefits to forage and livestock production in this environment.

## Supporting information

S1 Dataset Supporting data for paper. (XLSX)

## References

[1] Allen VG, Batello C, Berretta EJ, Hodgson J, Kothmann M, Li X, et al An international terminology for grazing lands and grazing animals. Grass and Forage Science. 2011;66(1):2–28.

[2] Earl JM, Jones CE. The need for a new approach to grazing management—is cell grazing the answer? Rangeland Journal 1996; 18:327–50.

[3] Jones CE, editor. Grazing management for healthy soils. Stipa Inaugural National Grasslands Conference ‘Better Pastures Naturally’; 2000; Mudgee, NSW Austrailia.

[4] Volesky JD, Schacht WH, Redden MD, Lindsey T, Johnson J, editors. Grazing strategy effects on herbage utilization, production, and animal performance on Nebraska Sandhills Meadow. 2016 Proceedings of the 10th International Rangeland Congress 2016.

[5] Savory A. Holistic resource management. Covelo, CA, USA: Island Press; 1988.

[6] Bisinger JJ, Russell JR, Bear DA, J. Sellers, Offenburger H. Enhancing botanical composition and wildlife habitat of pastures in south central iowa through soil disturbance by mob-grazing of beef cattle 2014. Animal Industry Report: AS 660, ASL R2888.

[7] Salatin J. An aggressive approach to controlled grazing: tall grass mob stocking. Acres USA 2008; Vol. 38, No. 5(No.5).

[8] Tietz N. Mob Grazing Produces Prime Pastures. Hay and Forage Grower. 2011 2 2, 2011.

[9] Bertelsen BS, Faulkner DB, Buskirk DD, Castree JW. Beef cattle performance and forage characteristics of continuous, 6-paddock, and 11-paddock grazing systems. J Anim Sci. 1993;71:1381–9. 10.2527/1993.7161381x 8392043

[10] Paine LK, Undersander D, Casler MD. Pasture growth, production, and quality under rotational and continuous grazing management. J Prod Agric 1999 12::569–77.

[11] Briske DD, Derner JD, Brown JR, Fuhlendorf SD, Teague WR, Havstad KM, et al Rotational grazing on rangelands: reconciliation of perception and experimental evidence. Rangeland Ecology and Management. 2008;61(1):3–17.

[12] Oates LG, Balser TC, Jackson RD. Sub-humid pasture soil microbial communities affected by presence of grazing, but not grazing management. Applied Soil Ecology. 2012;59(0):20–8.

[13] Shenk J, Workman J, Westerhaus M. Applications of NIR spectroscopy to agricultural products In: Burns D, Ciurczak E, editors. Handbook of Near-Infrared Analysis. Boca Raton, FL: CRC Press; 2007 p. 348–82.

[14] Eversole D, Browne M, Hall J, Dietz R. Body condition scoring beef cows. Virginia Cooperative Extension Publication. 400–791; 2009.

[15] Daubenmire RF. A canopy-cover analysis of vegetation analysis. Northwest Science. 1959;33:43–6.

[16] Tracy BF, Faulkner DB. Pasture and Cattle responses in rotationally stocked grazing systems sown with differing levels of species richness. Crop Sci. 2006;46(5):2062–8.

[17] R. version 3.2.1 (2015-06-18) — "World-Famous Astronaut" Copyright (C) 2015 The R Foundation for Statistical Computing. 2015.

[18] Belesky D, Fedders J. Defoliation effects on seasonal production and growth rate of cool-season grasses Agron J. 1994;86:38–45.

[19] Ferraro DO, Oesterheld M. Effect of defoliation on grass growth. A quantitative review. Oikos. 2002;98(1):125–33.

[20] Bryan WB, Prigge EC. Grazing initiation date and stocking rate effects on pasture productivity. Argon J. 1994;86(1):55–8.

[21] Collins M, Fritz JO. Forage Quality In: Barnes RF, Nelson CJ, Collins M, Moore KJ, editors. Forages: An Introduction to Grassland Agriculture. Volume I 6th Edition ed Ames, IA: Iowa State University Press; 2003 p. 363–91.

[22] Bauer R. Mob stocking effects on herbage nutritive value, herbage accumulation, and plant species composition. MS Thesis. Blacksburg, Va.: Virginia Tech; 2015. https://www.semanticscholar.org/paper/Mob-stocking-effects-on-herbage-nutritive-value%2C-Bauer/24e30d1faf60300275b4acd540135906a1d0e7fb

[23] Whetsell MS, Rayburn EB, Osborne PI. Evaluation in Appalachian pasture systems of the 1996 (update 2000) National Research Council model for weaning cattle. J Anim Sci. 2006;84(5):1265–70. 10.2527/2006.8451265x 16612031

[24] Jones GB, Tracy BF. Pasture soil and herbage nutrient dynamics through five years of rotational stocking. Crop Sci. 2014;54(5):2351–61.

[25] Sollenberger LE, Vanzant ES. Interrelationships among forage nutritive value and quantity and individual animal performance. Crop Sci. 2011;51(2):420–32.

[26] Walker JW, Heitschmidt RK, Dowhower SL. Some effects of a rotational grazing treatment on cattle preference for plant communities. J Range Manage. 1989;42:143–8.

[27] Jung HG, Rice RW, Koong LJ. Comparison of heifer weight gains and forage quality for continuous and short-duration grazing systems. J Range Manage. 1985;38:144–8.

[28] Janssen L, McMurtry B, Stockton M, Smart A, Clay S. An economic analysis of high-intensity, short-duration grazing systems in South Dakota and Nebraska. Selected paper 2015 Agricultural & Applied Economics Association and Western Agricultural Economics Association Annual Meeting; San Francisco, CA 2015.

[29] Bartimus HL, Montgomery TG, Philipp D, Cater J, Coffey KP, Shanks BC. 118 Mob grazing effects on cattle performance in southeast Arkansas. J Anim Sci. 2016;94(suppl_2):55-.

[30] Pavlů V, Hejcman M, Pavlů L, Gaisler J, Hejcmanová-Nežerková P, Meneses L. Changes in plant densities in a mesic species-rich grassland after imposing different grazing management treatments. Grass and Forage Science. 2006;61(1):42–51.

[31] Black AD, Laidlaw AS, Moot DJ, O’Kiely P. Comparative growth and management of white and red clovers. Irish Journal of Agricultural and Food Research. 2009;48(2):149–66.

[32] Zegler CH, Brink GE, Renz MJ, Ruark MD, Casler MD. Management Effects on Forage Productivity, Nutritive value, and legume persistence in rotationally grazed pastures. Crop Sci. 2018;58(6):2657–64.

[33] Wiersma DW, Smith RR, Sharpee DK, Mlynarek MJ, Rand RE, Undersander DJ. Harvest Management effects on red clover forage yield, quality, and persistence. J Prod Agric. 1998;11(3):309–13.

[34] Sheaffer CC, Miller DW, Marten GC. Grass dominance and mixture yield and quality in perennial grass-alfalfa mixtures. J Prod Agric. 1990;3(4):480–5.

[35] Tracy BF, Schlueter DH, Flores JP. Conditions that favor clover establishment in permanent grass swards. Grassland Science. 2015;61(1):34–40.

[36] Schlueter D, Tracy B. Sowing method effects on clover establishment into permanent pasture. Agron J. 2012;104(5):1217–22.

